# Prediction of risk factors associated with the development of multidrug-resistant tuberculosis in patients with tuberculosis

**DOI:** 10.3389/fpubh.2025.1588196

**Published:** 2025-07-22

**Authors:** Haiyan Chen, Zhaowei Tong, Jianfeng Zhong, Yong Tong, Qingqiu Zeng, Bin Shen, Qun Song, Fuchu Qian, Xin Xiao

**Affiliations:** ^1^Department of Infection, Huzhou Central Hospital, Huzhou, Zhejiang, China; ^2^Department of Clinical Medicine, Huzhou University, Huzhou, Zhejiang, China; ^3^Huzhou Key Laboratory of Precision Medicine Research and Translation for Infectious Diseases, Huzhou Central Hospital, Huzhou, Zhejiang, China

**Keywords:** risk factors, multidrug-resistant tuberculosis, tuberculosis, prediction, predictive model

## Abstract

**Objective:**

This study aimed to develop and validate a reliable nomogram based on clinical factors to predict development of multidrug-resistant tuberculosis (MDR-TB) associated with individuals with tuberculosis (TB), so as to reduce the harm and economic burden caused by disease progression.

**Methods:**

The study included 4,251 individuals with TB who received treatment at Huzhou Central Hospital between January 2016 and December 2023, excluding 87 individuals because of infection with non-TB mycobacterium or incomplete information (including missing laboratory or clinical data). A total of 4,164 individuals (2,261 sputum smear-positive and 1,903 sputum smear-negative patients) were ultimately included in the analysis. This analysis incorporated clinical baseline presentations, demographic information, imaging findings, laboratory results, and clinical diagnoses to develop a predictive model for MDR-TB.

**Results:**

This study demonstrated that sex, age, a history of anti-TB therapy, body mass index (BMI) ≤ 18.5, smoking history, occupation, previously diagnosed TB, pulmonary cavitation, comorbidities, poverty, and C-reactive protein (CRP) ≥ 37.3 mg/L were major risk factors for MDR-TB in patients with TB. The area under the receiver operating characteristic (ROC) curve was 0.902 for the training group and 0.909 for the validation group. Calibration curve analysis revealed that the predicted and actual incidence rates of MDR-TB in the two groups were in good agreement, whereas decision curve analysis (DCA) indicated that the predictive model resulted in better clinical benefit.

**Conclusion:**

The nomogram model established in this study included 11 clinical characteristics and demographic features of patients with TB, which may be valuable for predicting MDR-TB.

## 1 Introduction

Tuberculosis (TB) is one of the most considerable infectious diseases and poses a severe threat to global public health. According to the 2024 World Health Organization Global TB Report (1), in 2023, TB was responsible for approximately 1.25 million deaths worldwide. The incidence of rifampicin-resistant TB/multidrug-resistant TB (MDR-TB) is estimated at approximately 400,000 new cases. TB has escalated to become the largest single-source infectious disease worldwide, causing almost twice as many deaths as Human Immunodeficiency Virus (HIV)/Acquired Immunodeficiency Syndrome (AIDS) (1).

China ranks fourth globally in terms of the burden of MDR-TB, accounting for approximately 7.3% of all MDR/rifampicin-resistant TB cases worldwide (1). MDR-TB significantly contributes to treatment failure in newly diagnosed TB and is associated with a high mortality rate. Early identification of drug resistance and timely, effective treatment are paramount in controlling MDR-TB (2, 3).

The continuous dissemination of MDR-TB poses one of the most serious and challenging barriers to global TB control efforts. Consequently, ongoing data collection and surveillance are essential to assess the risk of MDR-TB transmission (4). The burden of drug-resistant TB (DR-TB) is considerably more severe than that of common TB, requiring longer treatment, more adverse effects, higher costs, and lower cure rates (5). Early screening of risk factors for DR-TB allows for the early detection of the propensity to develop DR-TB and facilitates timely diagnosis and treatment (6). A study conducted by de Dieu Longo et al. (7) in the Central African Republic found that direct contact with a person who had been treated for TB, male sex, peri-urban/urban residence, and the presence of MDR-TB in the family were associated with the occurrence of MDR-TB. Likewise, Baya et al. (8) reported that factors, such as age ≤40 years, failed or retreated TB treatment, sputum smear findings of 3 + or more bacilli, and a history of contact with a patient with TB, were relevant to the diagnosis of MDR-TB. Additionally, a study conducted by Ma et al. (9) on patients with TB in northwestern China found that age >40 years, male sex, and retreated TB were risk factors for MDR-TB. Furthermore, a study from Luoyang, a city in central China, discovered that MDR-TB was significantly related to male sex, a history of treatment for TB, age ≤50 years, and residence in urban areas (10). Patients with TB admitted to Huzhou Central Hospital between January 2016 and December 2023 were included in this study. This study identified potential risk factors for the progression of MDR-TB in patients with TB and established a nomogram model to aid clinical practice and the development of accurate prevention and control strategies.

## 2 Materials and methods

### 2.1 Data collection and ethics

**Figure 1:**
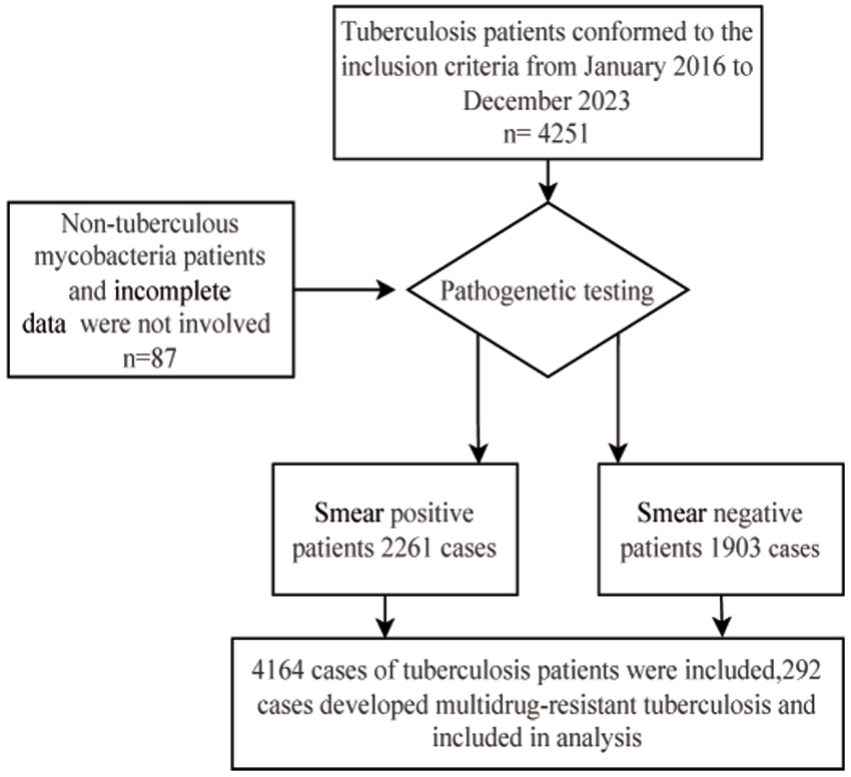
Flowchart of the study cohort.

Patients diagnosed with TB between January 2016 and December 2023 were included. Patients who underwent TB T-SPOT, sputum testing, or radiological examination were classified. The inclusion criteria were: (1) Polymerase Chain Reaction (PCR)-positive or culture-positive for *Mycobacterium tuberculosis* in sputum or bronchoalveolar lavage fluid; (2) patients with sputum-negative TB who had a history of close contact with infectious TB cases and showed corresponding clinical symptoms (including cough, productive cough, night sweats, low-grade fever), or those who were found to have abnormal shadows or active TB lesions on radiological examination and did not show any improvement after 2–4 weeks of TB treatment for common bacterial pneumonia. The exclusion criteria were: (1) incomplete information (such as missing laboratory or clinical data) and (2) infection with non-TB mycobacteria. A total of 4,251 patients with TB were enrolled in the cohort. After excluding 87 individuals because of infection with non-TB mycobacteria or incomplete data, 4,164 patients (including 2,261 sputum smear-positive and 1,903 sputum smear-negative patients with TB) were ultimately included in the analysis (Figure 1). The clinical data related to these cases were collected from the hospital’s electronic medical record system and infectious disease reporting network. The collected information included: (1) demographic characteristics (sex, age, occupation, hygienic conditions, and poverty) and (2) other factors: history of TB treatment, comorbidities, residence, sputum smear or molecular biology results, *Mycobacterium tuberculosis* culture results, and molecular drug sensitivity results.

These individuals were then randomly assigned in a 7:3 ratio to the training (*n* = 2,915) and validation (*n* = 1,249) groups. The training group consisted of 204 patients with MDR-TB and 2,711 patients with non-MDR-TB, whereas the validation group consisted of 88 patients with MDR-TB and 1,161 patients with non-MDR-TB. The study was approved by the hospital’s medical ethics committee (approval number: 2020-1020-01), and retrospective anonymized data collection methods were used to protect patient privacy. All experiments were conducted in accordance with the relevant laws, guidelines, and ethical standards of the Declaration of Helsinki.

### 2.2 Definitions of drug resistance

*DR-TB* was defined as TB resistant to at least one anti-TB drug. MDR-TB was defined as TB resistant to at least rifampicin and isoniazid. XDR-TB was defined as resistance to at least one fluoroquinolone and one of three second-line injectable drugs (capreomycin, kanamycin, and amikacin), in addition to MDR-TB (3).

*Initial treated TB*: Individuals who have not received anti-TB medications, those who have not completely undergone the standardized treatment regimen, and those who have experienced irregular therapy for less than a month.

*Retreated TB*: Individuals who received inappropriate and irregular anti-TB treatment for a minimum of 1 month, as well as those with treatment failure and relapse.

*Etiology positive*: Positive results characterized by sputum-based and/or molecular biology-based confirmation.

*Sputum positive*: Sputum or bronchoalveolar lavage smear positive for acid-fast bacilli and *Mycobacterium tuberculosis* cultures.

(1) Sputum or bronchoalveolar lavage smear results positive for acid-fast bacilli and/or *Mycobacterium tuberculosis*.
(2) *Mycobacterium tuberculosis* culture results.

Molecular biology positive:

(1) Xpert MTB/RIF positive.
(2) Targeted high-throughput sequencing positive.

*Etiology negative*: No evidence of *Mycobacterium tuberculosis* infection found through sputum or bronchoalveolar lavage fluid smears, *Mycobacterium tuberculosis* culture, Gene Xpert testing, metagenomic sequencing, or other methods.

*Sputum negative*: Sputum or bronchoalveolar lavage smear negative for acid-fast bacilli, with no positive *Mycobacterium tuberculosis* cultures.

### 2.3 Statistical analysis

All statistical analyses were performed using Free Statistics software version 2.0. Continuous variables were tested for normality, and normally distributed variables were expressed as mean and standard deviation (SD). Categorical variables were described using frequencies and proportions.

We used the least absolute shrinkage and selection operator (LASSO) regression to determine potential predictor variables for MDR-TB occurrence and conducted multivariate logistic regression analyses using selected features from the LASSO regression to identify statistically significant predictor variables. These were then used in the construction of a nomogram.

The area under the curve (AUC) was applied to assess the discriminatory power of the nomograms. Calibration of the nomograms was assessed using the Hosmer–Lemeshow test. Internal validation was performed using the bootstrap method, wherein 1,000 samples were randomly selected from the raw data for bootstrap replications. Calibration AUC values were calculated, and calibration curves were plotted to assess the predictive ability of the nomogram. Decision curve analysis was used to assess the net clinical benefit.

## 3 Results

### 3.1 Characteristics of the study population

Among the TB population, 7.01% (292/4,164) had MDR-TB. Most patients with TB had a body mass index (BMI) greater than 18.5 (91.5%), were farmers (79.3%), smokers (71.9%), men (69.6%), and resided in rural areas (61.9%). Regarding treatment, 91.1% of the patients were initially treated, and 8.9% were retreated. Among the MDR-TB population, 76.4% were men and 23.6% were women, 49% were ≤50 years of age, and 51% were >50 years of age. Most patients with MDR-TB were new cases (89.4%), had a smoking history (86%), had a BMI > 18.5 (84.2%), were farmers (83.6%), had CRP > 37.3 (54.5%), and had pulmonary cavitation (52.1%). Among patients with TB having comorbidities, 59.5% (287/482) had diabetes, 27.2% (131/482) had a history of pneumoconiosis, and 7.7% (37/482) had HIV. Sputum smear results were positive in 54.3% of TB cases and in 82.5% of MDR-TB cases. The additional baseline data are presented in Table 1.

**Table 1:** Clinical and demographic characteristics of tuberculosis and multidrug-resistant tuberculosis.

Variables	Total (*n* = 4,164)	TB (*n* = 3,872)	MDR-TB (*n* = 292)	*P*
Sex, n (%)				0.01
Men	2,900 (69.6)	2,677 (69.1)	223 (76.4)	
Women	1,264 (30.4)	1,195 (30.9)	69 (23.6)	
Age, n (%)				0.958
≤ 50	2033 (48.8)	1890 (48.8)	143 (49)	
>50	2,131 (51.2)	1982 (51.2)	149 (51)	
Residence, n (%)				0.831
Urban	1,587 (38.1)	1,474 (38.1)	113 (38.7)	
Rural	2,577 (61.9)	2,398 (61.9)	179 (61.3)	
Sputum smear, n (%)				< 0.001
Negative	1903 (45.7)	1852 (47.8)	51 (17.5)	
Positive	2,261 (54.3)	2020 (52.2)	241 (82.5)	
Type of TB treatment, n (%)				0.288
New cases	3,793 (91.1)	3,532 (91.2)	261 (89.4)	
Retreated cases	371 (8.9)	340 (8.8)	31 (10.6)	
Poverty, n (%)				< 0.001
No	3,257 (78.2)	3,000 (77.5)	257 (88)	
Yes	907 (21.8)	872 (22.5)	35 (12)	
Hygienic condition, n (%)				0.092
Well	2,928 (70.3)	2,710 (70)	218 (74.7)	
Poor	1,236 (29.7)	1,162 (30)	74 (25.3)	
Smoking history, n (%)				< 0.001
No	1,170 (28.1)	1,129 (29.2)	41 (14)	
Yes	2,994 (71.9)	2,743 (70.8)	251 (86)	
Drinking history, n (%)				0.012
No	3,410 (81.9)	3,155 (81.5)	255 (87.3)	
Yes	754 (18.1)	717 (18.5)	37 (12.7)	
Pulmonary cavity, n (%)				< 0.001
No	3,337 (80.1)	3,197 (82.6)	140 (47.9)	
Yes	827 (19.9)	675 (17.4)	152 (52.1)	
BMI, n (%)				< 0.001
≤ 18.5	352 (8.5)	306 (7.9)	46 (15.8)	
>18.5	3,812 (91.5)	3,566 (92.1)	246 (84.2)	
CRP, n (%)				< 0.001
<37.3	3,511 (84.3)	3,378 (87.2)	133 (45.5)	
≥37.3	653 (15.7)	494 (12.8)	159 (54.5)	
Occupation, n (%)				0.062
Farmer	3,302 (79.3)	3,058 (79)	244 (83.6)	
Others	862 (20.7)	814 (21)	48 (16.4)	
Comorbidities, n (%)				< 0.001
No	3,682 (88.4)	3,532 (91.2)	150 (51.4)	
Yes	482 (11.6)	340 (8.8)	142 (48.6)	
Lung diseases history, n (%)				< 0.001
No	3,371 (81.0)	3,183 (82.2)	188 (64.4)	
Yes	793 (19.0)	689 (17.8)	104 (35.6)	
WBC, Mean ± SD	6.5 ± 2.0	6.5 ± 2.0	6.6 ± 1.9	0.234
HGB, Mean ± SD	135.2 ± 17.4	135.2 ± 17.3	135.1 ± 18.4	0.935
PLT, Mean ± SD	188.6 ± 50.8	188.3 ± 51.1	193.4 ± 46.5	0.095
ALT, Mean ± SD	16.2 ± 6.3	16.0 ± 6.3	18.0 ± 7.2	< 0.001
AST, Mean ± SD	14.1 ± 5.2	14.0 ± 5.1	16.2 ± 5.7	< 0.001
Cr, Mean ± SD	69.5 ± 12.1	69.3 ± 11.9	72.4 ± 14.2	< 0.001

TB, tuberculosis; MDR-TB, multidrug-resistant tuberculosi; CRP, C-reactive protein; WBC, white blood cell; HGB, hemoglobin; PLT, platelet; ALT, alanine aminotransferase; AST, Aspartate aminotransferase; Cr, creatinine.

**Figure 2:**
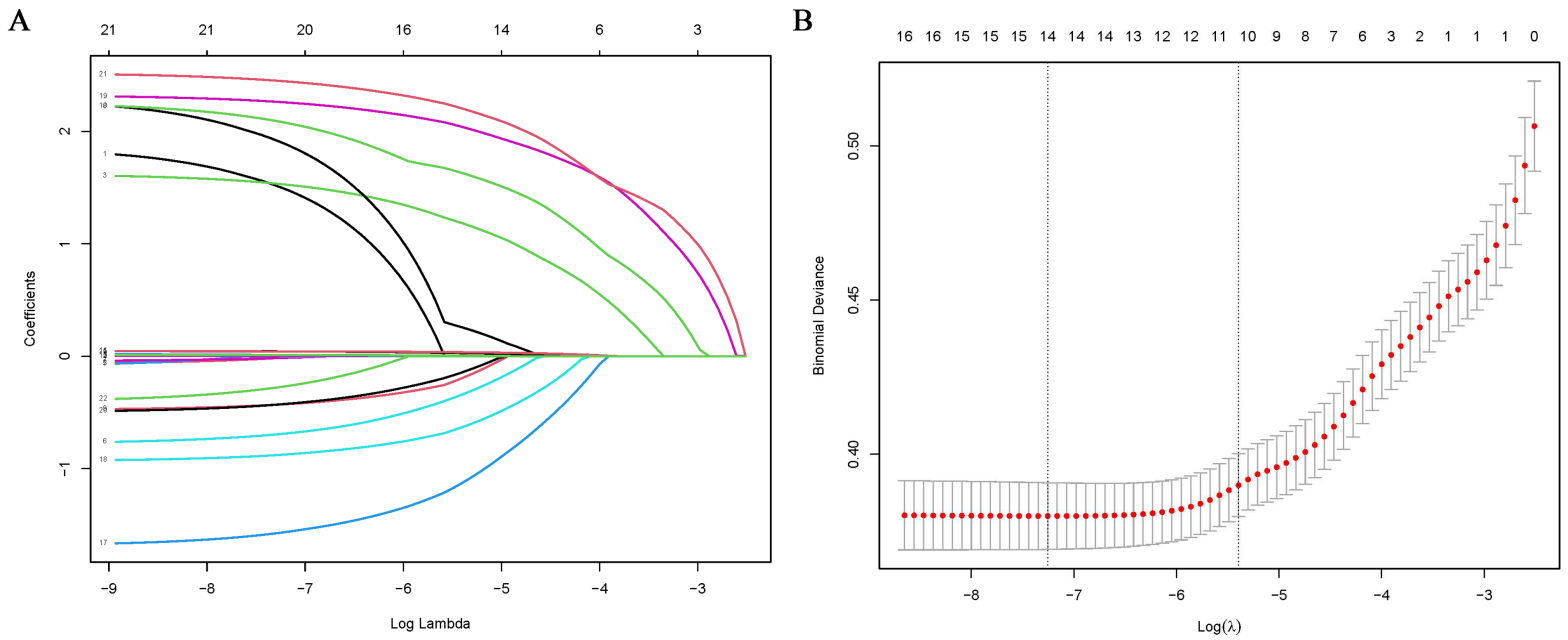
Feature selection using LASSO binary logistic regression model. **(A)** Log (lambda) value of 21 features in the LASSO model. A coefficient profile plot was produced against a log (lambda) sequence. **(B)** Parameter selection in the LASSO model uses five-fold cross-validation through minimum criterion. Partial likelihood deviation (binomial deviation) curves and logarithmic (lambda) curves are plotted. Minimum standard and 1-SE of the minimum standard are used to draw a vertical dashed line at the optimal value. Optimal lambda produces 15 nonzero coefficients. LASSO, least absolute shrinkage and selection operator.

Eleven of the 21variables in the clinical collection were selected based on nonzero coefficients calculated from the LASSO regression analysis (Figure 2). These variables included sex, age, type of TB treatment, BMI, smoking history, occupation, lung disease history, pulmonary cavitation, comorbidity, poverty, and CRP level.

### 3.2 Multivariate analysis of MDR-TB in patients with TB

Multivariate logistic regression analysis was performed to identify the risk factors for MDR-TB. The analysis was conducted while adjusting for confounding variables. The results showed that the risk of progressing to MDR-TB was 8.23 times higher in those with CRP ≥ 37.3 mg/L than in those with CRP < 37.3 mg/L [Odds Ratio (OR) = 8.23, 95% confidence interval (CI): 6.36–10.64, *p* < 0.001]. Moreover, the risk of developing MDR-TB was 7.21 times higher in patients with pulmonary cavitation than in those without (OR = 7.21, 95% CI: 5.39–9.64, *p* < 0.001). Furthermore, the risk of developing MDR-TB in individuals with comorbidities was 5.72 times higher than in individuals without comorbidities (OR = 5.72, 95% *CI*: 3.51–9.34, *p* < 0.001). Additionally, the risk of MDR-TB in individuals with a history of lung disease was 2.96 times higher than in those without a history of lung disease (OR = 2.96, 95% *CI*: 2.22–3.93, *p* < 0.001). Finally, the risk of developing MDR-TB in patients with a BMI > 18.5 was 0.42 times that of patients with a BMI ≤ 18.5 (OR = 0.42, 95% *CI*: 0.29–0.59, *p* < 0.001). The additional multivariate logistic regression analysis data are presented in Table 2.

**Table 2:** Multivariate logistic regression analysis of MDR-TB individuals with TB.

Variable	Total	n (%)	OR (95%CI)	*P*
Sputum smear, n (%)				
Negative	1903	51 (2.7)	1(Ref)	
Positive	2,261	241 (10.7)	4.48 (3.27 ~ 6.16)	<0.001
Sex, n (%)				
Men	2,900	223 (7.7)	1(Ref)	
Women	1,264	69 (5.5)	0.62 (0.46 ~ 0.82)	0.001
Type of TB treatment, n (%)				
New cases	3,793	261 (6.9)	1(Ref)	
Retreated cases	371	31 (8.4)	1.23 (0.83 ~ 1.82)	0.31
Smoking history, n (%)				
No	1,170	41 (3.5)	1(Ref)	
Yes	2,994	251 (8.4)	2.96 (2.09 ~ 4.2)	<0.001
Pulmonary cavity, n (%)				
No	3,337	140 (4.2)	1(Ref)	
Yes	827	152 (18.4)	7.21 (5.39 ~ 9.64)	<0.001
Age, n (%)				
≤50	2033	74 (3.6)	1(Ref)	
>50	2,131	73 (3.4)	0.94 (0.68 ~ 1.31)	0.708
BMI, n (%)				
≤18.5	352	46 (13.1)	1(Ref)	
>18.5	3,812	246 (6.5)	0.42 (0.29 ~ 0.59)	<0.001
CRP, n (%)				
<37.3	3,511	133 (3.8)	1(Ref)	
≥37.3	653	159 (24.3)	8.23 (6.36 ~ 10.64)	<0.001
Comorbidities, n (%)				
No	3,682	150 (4.1)	1(Ref)	
Yes	482	142 (29.5)	5.72 (3.51 ~ 9.34)	<0.001
Lung diseases history, n (%)				
No	3,371	188 (5.6)	1(Ref)	
Yes	793	104 (13.1)	2.96 (2.22 ~ 3.93)	<0.001

TB, tuberculosis; MDR-TB, multidrug-resistant tuberculosi; BMI, Body Mass Index; CRP, C-reactive protein.

### 3.3 Constructing and evaluating the predictive model

**Figure 3:**
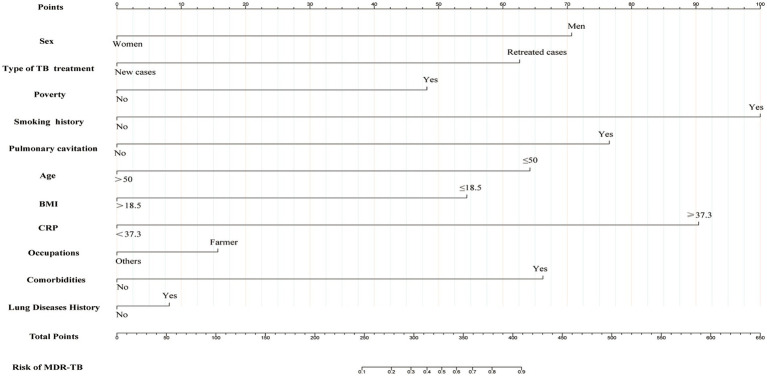
Nomogram for predicting the risk of development of MDR-TB in individuals with TB. The first line represents the scoring scale. Corresponding scores for each predictor factor are shown in lines 2–12. The score for each predictor is determined by referencing the first line. The total score for the risk evaluation is the sum of each predictor score. To determine the likelihood of the above risk factors contributing to MDR-TB, the score point is located on the total point line (line 13). Then, the user descends vertically to the MDR-TB risk (line 14). With a higher score indicating a higher risk of MDR-TB.

To construct a predictive model for MDR-TB, we performed multivariate logistic regression analysis based on the 11 variables (sex, age, type of TB treatment, CRP, BMI, smoking history, occupation, lung disease history, pulmonary cavitation, comorbidity, and poverty) selected using the LASSO regression technique. There was no prominent correlation among these factors (*R*^2^ = 0.468). These independent predictors were thus incorporated to develop a predictive nomogram (Figure 3), with a higher score indicating a higher risk of MDR-TB. Men, retreated cases, poor, history of smoking, pulmonary cavity, age ≤ 50 years, BMI ≤ 18.5, CRP ≥ 37.3, farmer, comorbidities, and lung disease history were risk factors for developing MDR-TB.

**Figure 4:**
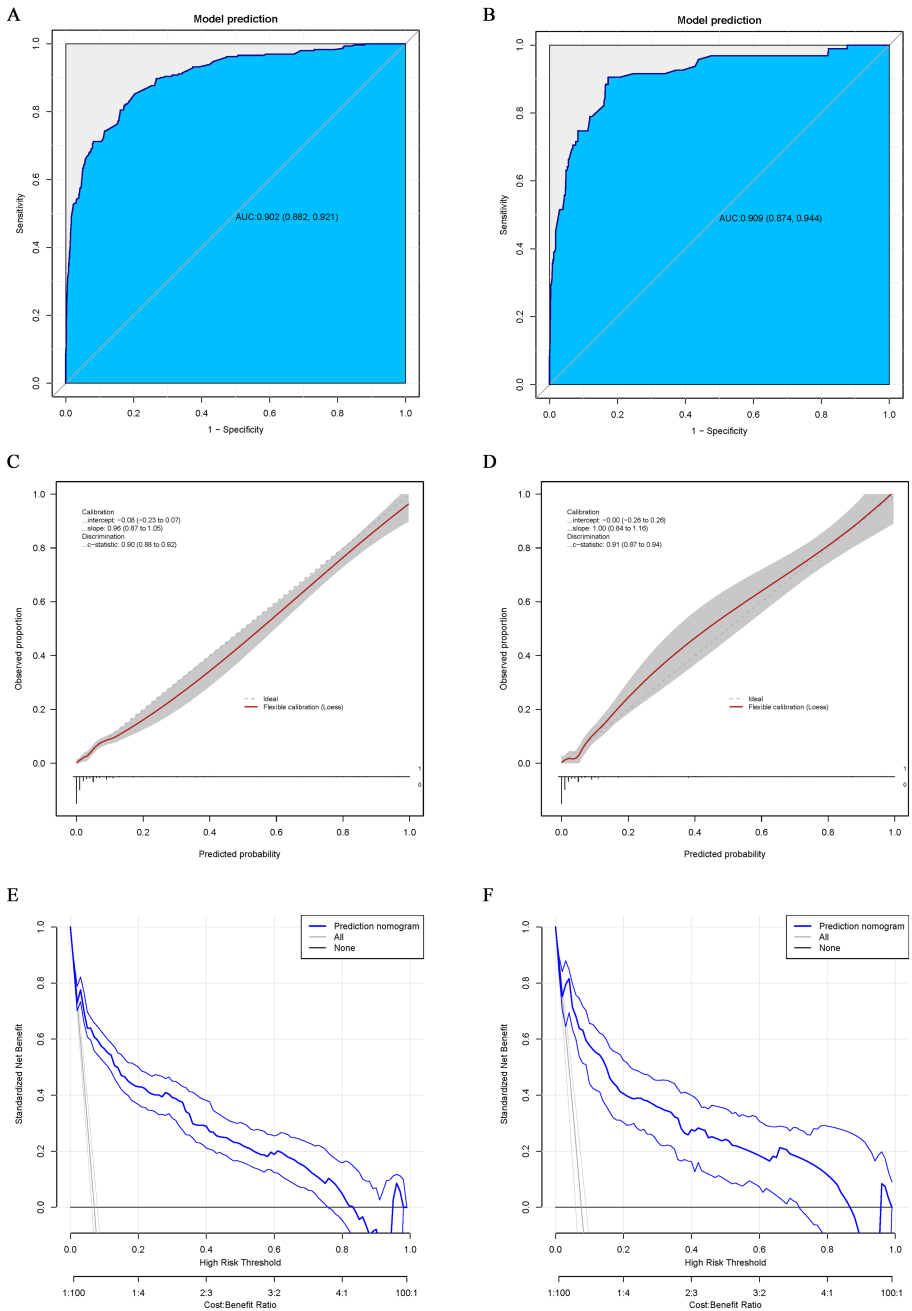
ROC curve for the nomogram of the training **(A)** and validation groups **(B)**. The point on the curve represents the optimal cutoff value (specificity, sensitivity). The brackets next to the area under the ROC curve (AUC) represent the 95% confidence interval. Calibration curve for the nomogram of the training **(C)** and validation groups **(D)**. The apparent curve represents the relationship between predicted and actual probabilities of the development of MDR-TB. The bias-corrected curve is plotted by bootstrapping using 1,000 resamples. The ideal curve is the 45° line, which indicates perfect prediction. Decision curve analysis (DCA) for the nomogram of the training **(E)** and validation groups **(F)**. Blue solid lines represent the nomogram, x axis, cutoff probability, and y axis, net benefit. AUC, area under the curve; ROC, receiver operating characteristic; DCA, Decision curve analysis.

The AUC of the training and validation groups was 0.902 (95% CI: 0.882–0.921) and 0.909 (95% CI: 0.874–0.944), respectively, indicating that the model had good discriminative ability (Figures 4A,B). Calibration curve analysis using the Bootstrap method illustrated that the calibration curves of both the training and validation groups were close to the standard curves. The Hosmer–Lemeshow test results indicated a good fit of the predictive model, with *p*-values of 0.90 and 0.91, respectively (Figures 4C,D. The decision curve analysis (DCA) results for both the training and validation groups indicated a high rate of net clinical benefit from the model (Figures 4E,F.

## 4 Discussion

The emergence and spread of MDR-TB is a persistent threat to global health security and presents a challenge to the “End TB by 2035” strategy. MDR-TB often results in protracted treatment, unfavorable reactions, increased costs, and poses challenges for managing relapses and difficult-to-treat cases (11). Therefore, enhanced surveillance of the burden is essential to assess risk, and urgent and targeted interventions are necessary to improve detection, treatment, and prevention strategies (4, 12). Moreover, precise diagnosis and treatment of MDR-TB should be available and accessible to all patients (11).

In this study, 11 variables (sex, age, type of TB treatment, CRP level, BMI, smoking history, occupation, lung disease history, pulmonary cavity, comorbidity, and poverty) were screened to predict the risk of MDR-TB using multivariate logistic regression analysis, combining clinical indicators and demographic information. Sex, age, residence, poverty, previous diagnosis of TB, smoking history, lung cavitation, history of anti-TB therapy, nationality, immigration status, and positive sputum smears are significant factors associated with the incidence of MDR-TB (13–16)^.^

This study discovered that the detection rate of DR-TB in women is 0.38 times lower than that in men. This suggests that men carry a heavier burden of DR-TB, potentially attributable to their lifestyle, working environment/occupation [exposure to higher levels of fine dust (17)], and smoking habits (18). A study exploring the association between smoking and DR-TB suggested that smoking is an independent risk factor for the development of DR-TB (19). The predictive modeling in this study also found that smoking history was a key predictor of the occurrence of DR-TB.

It is well known that patients with TB who have been retreated for TB are more susceptible to progress toward MDR-TB than those who were initially treated. Global studies conducted in Malaysia, Central Africa, China, and India have supported this finding (7, 9, 13, 20).

The rationale for this conclusion is that patients with TB who have been retreated may not be able to fully suppress MTB because of inadequate or unstandardized prior anti-TB treatment, which may result in some MTB strains adapting to the drug environment and developing resistance. In addition, if patients with TB stop taking their medication after the symptoms disappear or because of poor compliance, this may lead to treatment failure or relapse, which can result in continued positive sputum cultures and persistent lesions, greatly increasing the possibility of secondary DR-TB. This finding emphasizes the significance of appropriate and effective treatment by healthcare providers and enhanced supervision and follow-up of patients with TB.

Clinically, a BMI < 18.5 kg/m^2^ was defined as malnutrition. In this study, BMI ≤ 18.5 was an apparent risk factor for predicting the occurrence of MDR-TB. A report on Indian patients with MDR-TB showed that BMI < 18 was remarkably associated with sputum culture reversion, sputum culture non-conversion, and/or poor treatment outcomes (21). Similarly, Bade et al. (22) indicated that delayed sputum culture conversion in patients with MDR-TB was related to malnutrition. Malnutrition is also a risk factor for poor outcomes with MDR-TB treatment (9, 22).

Pulmonary cavity is a common clinical manifestation in immunocompetent patients with TB. However, cavity may not occur in immunocompromised patients. The presence of lung cavitation often indicates severe lung damage and is a risk factor for MDR-TB development. Xi et al. (3) performed a global meta-analysis and observed a significant correlation between lung cavitation and MDR-TB. The existence of lung cavitation not only provides an environment for the survival and replication of MTB but may also increase the risk of MDR-TB by contributing to its adaptation and tolerance to anti-TB drugs. It also prevents full penetration of anti-TB drugs into the affected area and reduces the effectiveness of treatment. In addition, pulmonary cavitation disrupts the local immune response, weakening the body’s ability to clear MTB. This increases the susceptibility of patients with TB to other drug-resistant strains and the development of MDR-TB.

In this study, patients with comorbidities were mainly diagnosed with diabetes, pneumoconiosis, and HIV, and individuals with TB in combination with these comorbidities had an increased likelihood of progressing to MDR-TB. One study provided evidence that patients with TB and diabetes are more likely to develop MDR-TB than non-diabetic patients with TB, and diabetes increases the risk of treatment failure in patients with TB (23). In addition, another study showed an increasing incidence of TB, including MDR-TB, and mortality owing to treatment failure in workers exposed to silica dust compared to their unexposed counterparts (24). HIV infection also increased the likelihood of MDR-TB. A report indicated that the risk appeared to be on the rise compared to an earlier pooled study (25).

Poverty is also an important risk factor for MDR-TB progression. Owing to poverty, many patients are not treated in a timely manner, their treatment is not standardized, or they lack the financial ability to pay for expensive treatments. This often results in treatment interruptions, leading to treatment failures and inducing drug resistance. Therefore, there is an urgent need for increased political commitment and resources to reduce poverty if MDR-TB control is to be realized (16).

Patients with TB had a greater likelihood of developing MDR-TB, consistent with the findings of Wu et al. (14). In this study, we developed and validated a nomogram model for predicting the risk of MDR-TB using clinical characteristics, demographic information, and laboratory and imaging indices of patients with TB. Higher AUC values indicate that the model has valid discriminatory power, Hosmer–Lemeshow test results demonstrate preferable calibration, and the DCA curve results suggest that the model has practical clinical usefulness. The model’s metrics are readily available in clinical settings and serve as practical tools for predicting the occurrence of MDR-TB. Screening risk factors through predictive modeling, enhancing screening of high-risk groups, and adopting effective treatment strategies to prevent disease progression can help interrupt the spread of the disease and reduce the burden on society.

In conclusion, this study constructed a model for predicting the risk of developing MDR-TB in patients with TB based on sex, age, type of TB treatment, CRP, BMI, smoking history, occupation, lung disease history, pulmonary cavity, comorbidity, and poverty. The model exhibited high accuracy and clinical utility, providing a useful reference for the effective diagnosis, treatment, prevention, and control of MDR-TB in clinical applications.

## 5 Limitations

However, this study also has several limitations. Firstly, as it was a retrospective study, the indicators were limited to the available variables, which may lead to bias. As the nutritional status of TB patients is very critical in the assessment of their disease progression or improvement, only the BMI of the patients was included in our evaluation indexes, and most of the patients were not included due to the lack of information on serum albumin, hemoglobin, micronutrients such as vitamin D, iron, and zinc, and the MUST (Malnutrition Universal Screening Tool) scores, and more evaluation indexes of the nutritional status will be collected and analyzed in the future. Some of the patients were retreatment patients with poor adherence, and some discontinued because of economic deprivation or low education, and these factors should be included in future cohort studies.

It is generally believed that the development of tuberculosis is the effect of genetic susceptibility and environmental factors, and that first-degree relatives of tuberculosis patients, such as parents and siblings, are more susceptible to the risk of the disease than the general population. The key gene mutations affect tuberculosis susceptibility, such as the HLA genes, the TLR genes, the IL-12/IL-23 pathway genes, and the genes for vitamin D metabolism, may increase the likelihood of tuberculosis and MDR-TB. In this study, the data on genetic susceptibility was lacked, and these information need to include as much as possible in future studies. Moreover, genetic diversity of TB strains (e.g., Beijing family, MDR-TB) profoundly affects disease transmission, clinical treatment. Drug resistance is still the biggest challenge, which needs to be combined with molecular diagnosis and monitoring to realize precise prevention and control. Mutations were detected in 78.33% (94/120) and 19.17% (23/120) of isoniazid resistance loci katG and inhA, respectively. Combined mutations in katG and inhA were relatively rare. Among the mutations associated with rifampicin resistance, rpoB531, rpoB526 and rpoB511 were the most common. The mutation rates of rpoB + katG and rpoB + inhA in multidrug-resistant *M. tuberculosis* strains were 73.44% (47/64) and 21.88% (14/64), respectively. However, there are still many patients with missing or incomplete information on the drug resistance mutation sites. In the future, we will try our best to collect more comprehensive strain characterization of multidrug-resistant patients. We will do more analysis of possible residual confounding by such as the uncollected variables mentioned above.

Secondly, the data were obtained from a single center, thus limiting their generalizability. In the future, we intend to further validate the established prediction model in the TB-related MDR-TB cohort dataset of Jiaxing First People’s Hospital and Hangzhou Red Cross Hospital. Meanwhile, the established prediction model was further validated the differentiation, calibration, and clinical applicability of the model in the TB-associated MDR-TB cohort dataset of TB patients attending our hospitals in the next 3 years to evaluate the practical application effect of the model.

Thirdly, the present lacked subgroup analysis and sensitivity analysis for different age subgroups, comorbidities, new cases and retreatment cases. In the future, we will compare the incidence of MDR-TB in different age group (<18 years old group, 18–65 years old group, and >65 years old group), and estimate the effect of weighting of different age groups on the incidence of MDR-TB. Furthermore, we will expand the cohort and conduct subgroup analyses and sensitization for comorbidities such as coinfected with HIV, diabetes, and pneumoconiosis, and explore any interactions and comorbidity-specific risk scores (e.g., adjusting weights for inflammatory markers in diabetic patients). History of prior treatment failure and baseline genotypic resistance (e.g., *rpoB* mutations) served as strong predictors of MDR-TB occurrence in retreatment cases. We will conduct subgroup analyses of primary and retreated cases to predict MDR-TB. There are still challenges in the diagnosis and treatment of TB patients and MDR-TB.

There is a large scale mobility in this region, and some patients are from western regions such as Yunnan, Guizhou and Sichuan, where are economically poorer and do not have Medical insurance, and some of the patients are traveling between two places, which can easily lead to interruption of the treatment, and the treatment information is not comprehensive. Considering the cost-effectiveness, genotyping testing has not yet been fully popularized. For these reasons, the Chinese Government has established territorial management measures that allow patients to be concentrated in designated hospitals as much as possible, while it can reduce the risk of transmission of tuberculosis due to population mobility.

In summary, successful implementation of the MDR-TB predictive model needs to address the three vital issues of data quality, model generalizability, and clinical application, and demonstrating its value through cost–benefit analysis. The key to health system integration is: Technology compatibility, systems integration, model clinical application guided by model action, multi-party collaboration (hospitals, CDC, and medical insurance department).

## Data Availability

The datasets presented in this study can be found in online repositories. The names of the repository/repositories and accession number(s) can be found in the article/Supplementary material.

## Glossary

TB: tuberculosis
MDR-TB: multidrug-resistant tuberculosis
DR-TB: drug-resistant tuberculosis
BMI: body mass index
CRP: C-reactive protein
ROC: receiver operating characteristic
DCA: decision curve analysis
AUC: area under the curve
LASSO: least absolute shrinkage and selection operator
PCR: Polymerase Chain Reaction
HIV: Human Immunodeficiency Virus
AIDS: Acquired Immunodeficiency Syndrome
SD: standard deviation
OR: Odds Ratio
CI: confidence interval
WBC: white blood cell
HGB: hemoglobin
PLT: platelet
ALT: alanine aminotransferase
AST: Aspartate aminotransferase
Cr: creatinine
